# Adding gastrointestinal parasite resistance to the breeding objective in hair sheep: initial steps

**DOI:** 10.1093/jas/skae327

**Published:** 2024-10-26

**Authors:** Robert M Forbes, Thomas W Murphy, Joan M Burke, David R Notter, Matthew L Spangler, Michael D MacNeil, Ronald M Lewis

**Affiliations:** Department of Animal Science, University of Nebraska-Lincoln, Lincoln, NE 68583, USA; USDA, ARS, Livestock Bio-Systems Research Unit, Roman L. Hruska U.S. Meat and Animal Research Center, Clay Center, NE 68933, USA; USDA, ARS, Dale Bumpers Small Farms Research Center, Booneville, AR 72927, USA; School of Animal Sciences, Virginia Tech, Blacksburg, VA, 24061, USA; Department of Animal Science, University of Nebraska-Lincoln, Lincoln, NE 68583, USA; Delta G, Miles City, MT 59301, USA; Agricultural Research Council, Animal Production, Irene, South Africa; Department of Animal Sciences, University of the Free State, Bloemfontein, South Africa; Department of Animal Science, University of Nebraska-Lincoln, Lincoln, NE 68583, USA

**Keywords:** fecal egg count, gastrointestinal parasite resistance, genetic response, selection index, sheep

## Abstract

The U.S. Maternal Hair Index was designed by the National Sheep Improvement Program (**NSIP**) to increase total weight of lamb weaned per ewe lambing (**TW**). Producers are interested in adding gastrointestinal nematode (**GIN**) resistance to this breeding objective since parasitism causes substantial economic losses. The NSIP provides producers with estimated breeding values (**EBV**) for postweaning fecal egg count (**PFEC**), an indicator of GIN resistance. Our objective was to gauge the effects of including PFEC as another selection criterion and goal trait. Selection index theory was used to construct 11 indexes. First was a benchmark index with TW as the goal. Weaning weight, and number of lambs born and weaned, were selection criteria. An index was then designed with PFEC added as a selection criterion. In 9 more indices, PFEC was also included as a goal trait, where the economic value of TW was scaled relative to that of PFEC. PFEC received a scaled economic value of −1 with that of TW increasing from +1 to +5 at +0.5 increments. Selection criteria were modeled as EBV or phenotypes. Annual genetic responses in goal traits were predicted. The top 3% of males and 26% of females were selected. Breeding values and phenotypes were simulated for 200 lambs by Cholesky decomposition and used to generate index scores, with 100 replicates run. Concordances with the animals selected in the benchmark scenario were determined. Using EBV as the selection criteria, TW increased by 1.46 kg/yr in the benchmark scenario. However, unfavorably, PFEC increased by 2.24%/yr. When using phenotypic criteria, TW increased by 0.52 kg/yr and PFEC by 0.28%/yr. Once added as a goal trait, PFEC decreased regardless of the scaled economic value of TW. However, responses in TW were also reduced, although less so as its scaled economic value increased. A scaled economic value of +3 for TW appeared reasonable over other choices with 79% of the emphasis placed on TW in the breeding objective. With EBV as selection criteria, PFEC declined by 7.96%/yr with 98% of the response in TW retained. Also, on average, 64% of males and 80% of females were chosen in common with the benchmark. With phenotypic selection criteria, PFEC declined by 5.13%/yr and 94% of the response in TW was retained; 61% of males and 80% of females were chosen in common with the benchmark. Implementing an index with scaled economic values of +3 for TW and −1 for PFEC would balance gains in TW with reductions in PFEC.

## Teaser

A selection index designed to achieve balanced genetic gains in total weight of lamb weaned per ewe lambing and gastrointestinal parasite resistance provides a simple selection tool for breeders to improve both traits simultaneously.

## Introduction

Since the introduction of the selection index theory in livestock by Hazel (1943), economic selection indices have generally been accepted as the best method to achieve genetic gain for multiple traits in animal breeding programs. The aim of these indexes is to raise overall animal productivity rather than improving just a single trait. Traditionally, breeders have placed emphasis on production traits in their indexes, while fitness traits, such as fertility, longevity, and health, have largely been ignored (Goddard, 1998). The sheep industry is no exception. For instance, indexes currently in use by the U.S. National Sheep Improvement Program (**NSIP**) include growth and carcass traits, wool production, and reproductive rate, but exclude other fitness traits such as disease resistance.

One of the breeds evaluated by NSIP is Katahdin, a relatively prolific maternal composite hair breed comparable to other medium-sized maternal breeds in adult bodyweight and lamb growth rates (Ngere et al., 2018). The breed also potentially exhibits greater resistance to gastrointestinal nematode (**GIN**) infection than other breeds that are common in the U.S. (Burke and Miller, 2004). The Katahdin recently became the breed with the most animals registered in the U.S. (Thorne et al., 2021), due perhaps in part because of its potential for GIN resistance.

Currently, the U.S. Maternal Hair Index (Vanimisetti et al., 2007), used by Katahdin and other hair sheep breeders, is based on 4 selection criteria: direct weaning weight, maternal weaning weight, number of lambs born (**NLB**), and number of lambs weaned (**NLW**). The breeding objective is to maximize the total weight of lamb weaned per ewe lambing (**TW**). Katahdin producers, however, have expressed interest in adding GIN resistance to the breeding objective. This has been demonstrated by their substantial industry-wide recording of fecal egg count (**FEC**), which are used as indicators of GIN infection. Including GIN resistance as a goal trait in the index also would generate a more comprehensive breeding objective (Notter et al., 2018). Such a change may be particularly advantageous for hair sheep enterprises because of their propensity to be located in climates where GIN thrive (Arisman et al., 2023a).

In the U.S., higher FEC have been associated with sheep that have larger litters and lower body weights (Ngere et al., 2018; Notter et al., 2018), are located in areas with hotter temperatures and greater rainfall, and have pasture-reared lambs (Arisman et al., 2023a). However, to measure the aggregate losses in profit due to morbidity from higher FEC in the U.S., additional information is needed to quantify its interactions with other aspects of production.

The emphasis placed on a trait in a selection index typically depends on its impact on net profit, which can be determined through a bioeconomic model or profit equation. Such strategies simulate the monetary effects on a production system when the flock mean for each trait is independently perturbed (MacNeil et al., 1997; Borg et al., 2007). However, given the challenges in defining the economic consequences of obtaining higher or lower FEC in individual flocks, the degree to which GIN resistance should be emphasized in an index for hair sheep breeds engaged in NSIP has not been well defined.

A substitute for determining the “true” economic value of a trait, such as FEC, is to evaluate its scaled economic value relative to the other traits in the breeding objective. This outcome can be achieved by assessing a range of possible scaled economic values and measuring their impact on predicted genetic gains for the individual goal traits. When balancing selection for carcass lean and fat weight, Simm and Dingwall (1989) observed that a scaled economic value of +3 for lean weight and −1 for fat weight nearly maximized gains in carcass lean weight while keeping increases in fat weight relatively small. Brien et al. (2020) adopted a similar approach when determining the economic value of breech flystrike in indexes for use in Australian Merino sheep. Economic values between −$60 to −$140/strike reduced the incidence of flystrike while maintaining reasonable responses for goal traits in the original index. Sivarajasingam (1995) estimated the scaled economic value of FEC relative to body weight, keeping the scaled economic value of body weight constant at +1 while varying that of FEC from −6 to +3. A favorable scaled economic value for FEC was then suggested for different breeding objectives by looking at the response curves of body weight and FEC. Similar methods could be used to find the scaled economic value for FEC in U.S. hair sheep through its addition to the breeding objective alongside TW.

The objective of this study was to examine predicted genetic responses in TW and postweaning FEC (**PFEC**) when PFEC was added to the breeding objective with varying emphasis. By quantifying changes in genetic responses across the range of scaled economic values tested, the scaled economic value for TW and PFEC that achieved acceptable rates of improvement in both traits should become evident. Additionally, the extent to which the alternative indexes affected candidates selected as replacements was assessed. The method adopted in this study is an ad hoc approach, which is non-optimal (Bourdon, 1998). However, it provided flexibility to investigate the consequences of including PFEC in the breeding objective on genetic response in TW.

## Materials and Methods

The data used to estimate the values of genetic and phenotypic parameters, construction of indexes, and simulation of index scores, were collected in Katahdin flocks enrolled in NSIP. Therefore, institutional animal care and use approval was not required.

### U.S. Maternal Hair Index

The breeding objective of the U.S. Maternal Hair Index is to predict TW (Vanimisetti et al., 2007). This prediction was calculated from the multiple linear regression of estimated breeding values (**EBV**) for TW on the EBV for its 4 component traits: direct weaning weight (**DWWT**), maternal weaning weight (**MWWT**), NLB, and NLW. With the introduction of genomic information to the genetic evaluation of hair sheep (McMillan et al., 2022), including re-estimation of the genetic and phenotypic parameter values used by NSIP, the index coefficients were updated in 2021. The index (*I*_TW_) equation currently in use is


$$\begin{array}{lll} I_{TW}= & \;100+(0.128EBV_{DWWT}+0.407EBV_{MWWT}\; & -4.401EBV_{NLB}+22.179EBV_{NLW}) \end{array}$$

where the numeric coefficients were again derived from the multiple linear regression of EBV for TW on the EBV for the 4 component traits.

### Selection criteria and goal trait

Among the selection criteria investigated were weaning weight (**WWT**), NLB, and NLW. Within NSIP, WWT (kg) are recorded in lambs at approximately 60 d of age. The NLB and NLW are collected repeatedly in retained ewes at each lambing.

A goal trait considered was TW (kg). As described by Vanimisetti et al. (2007), it reflects the combined effects of reproduction and preweaning growth and is calculated as the sum of the weaning weights of all lambs in the litter. When obtaining TW, WWT is adjusted to 60 d of age and for the effects of sex (to a ewe lamb basis) using multiplicative adjustment factors derived from Katahdin NSIP records (Notter and Kuehn, 2003).

Both EBV and phenotypes for the selection criteria were used when designing indexes. As noted earlier, in the genetic evaluation by NSIP, WWT is partitioned into DWWT and MWWT EBV. However, a phenotype for MWWT cannot be recorded. Therefore, to allow fair comparisons between indexes based on EBV and phenotypic selection criteria, direct and maternal EBV were considered together as a single amalgamated trait in the current study. This amalgamation corresponded with the sum of the EBV for DWWT and one-half the EBV for MWWT.

Since lambs were assumed to be selected prior to sexual maturity, they would not have had the opportunity to express their own reproductive capabilities. Therefore, lambs’ own birth and rearing types were considered as their pseudo-phenotypes for NLB and NLW, respectively, for indexes based on phenotypic selection criteria.

### PFEC as additional criteria and goal trait

PFEC (eggs/g) was considered as an additional selection criterion and goal trait. In NSIP, PFEC are recorded in lambs postweaning at approximately 90 d of age.

To consider the potential value of including PFEC in the breeding objective, a total of 11 indexes were created. The first index had TW as the breeding objective. Index coefficients were calculated using WWT, NLW, and NLB as selection criteria. This generated an index that emulated the current breeding objective of the U.S. Maternal Hair Index and served as a benchmark for comparison with other indexes. PFEC was excluded as a selection criterion and goal trait. However, correlated genetic response for PFEC was still predicted by including it in the breeding objective with a scaled economic value of zero.

A second index was then developed that kept TW as the sole breeding objective but added PFEC as a further selection criterion. Similar to the first index, correlated genetic response for PFEC was predicted by including it in the breeding objective with a scaled economic value of zero. Thereafter, 9 additional indexes were developed with both PFEC and TW in the breeding objective with PFEC continuing to be included as a selection criterion. In these 9 indexes, the scaled economic value of TW increased from +1 to +5 at +0.5 increments while the scaled economic value of PFEC remained constant at −1.

All 11 indexes were calculated using both EBV and phenotypes as selection criteria. The intent of this was to describe upper (EBV as criteria) and lower (phenotypes as criteria) bounds of expected genetic responses and accuracy from selection on the index.

### Parameter values

The genetic and phenotypic (co)variances used to obtain selection index coefficients and accuracies, and to predict genetic responses in goal traits, were provided by NSIP. The genetic variance for WWT (2.028 kg^2^) was approximated as the sum of its direct additive (1.521 kg^2^) and one-half its maternal additive (1.014 kg^2^) variance (Willham, 1972); the direct-maternal additive covariance for WWT is assumed to be zero in the NSIP genetic evaluation.

TW is not among the traits reported in the NSIP routine genetic evaluation. Therefore, estimates of the phenotypic and genotypic variances for TW, and its corresponding covariances with WWT, NLB, NLW, and PFEC, were combined with the other parameter values to form genetic and phenotypic (co)variances matrices for all 5 traits.

As often is the case, the parameter values were not estimated simultaneously in a multi-trait model. When combined they, therefore, may have formed (co)variance matrices that were not positive definite, which risked the correlation between the index and the breeding objective, or the index accuracy, exceeding unity (Meuwissen and Kanis, 1988). The eigenvalues and eigenvectors of the genetic and phenotypic (co)variances matrices were, therefore, obtained to determine if these matrices were consistent (positive definite). Although the phenotypic (co)variance matrix was positive definite, the genetic (co)variance matrix was not. For this 5 × 5 genetic (co)variance matrix, the eigenvalues were 9.836, 5.331, 0.863, 0.018, and −0.006.

Following the methodology of Hayes and Hill (1981), although applied to the genetic (co)variance matrix itself as recommended by Meuwissen and Kanis (1988), the estimated genetic parameter set was modified based on bending theory. Only a small bending factor (0.002) was needed to generate a positive definite matrix. The eigenvalues of the bent matrix were 9.835, 5.331, 0.863, 0.018, and 0.001, quite similar to those of the original matrix.

Estimated and bent genetic parameter values are provided in Table 1. Generally, the modified values were similar to the estimated values (less than a 3% change). The exceptions were the genetic variances of NLB and NLW, which increased by 26% and 34%, respectively. That coincided, however, with only a small change in the absolute magnitude of these variances (0.006 count^2^). Since the genetic covariance between NLB and NLW was unchanged by the bending procedure, the genetic correlation between NLB and NLW decreased from 0.80 to 0.62.

**Table 1.** Estimated and bent^1^ additive genetic ($\sigma_{a}^{2}$) variances, heritabilities, and correlations^2^

	$\sigma_{a}^{2}$						
Trait^3^	Estimated	Bent	TW	WWT	NLB	NLW	PFEC
TW	8.622 (1.368)	8.611	**0.072** (0.012)	0.723 (0.139)	0.481 (0.083)	0.854 (0.031)	−0.050 (0.158)
WWT	2.028 (0.259)	2.030	0.722	**0.172** (0.021)	0.199 (0.067)	0.130 (0.089)	−0.001 (0.105)
NLB	0.025 (0.004)	0.031	0.428	0.177	**0.074** ^4^ (0.010)	0.799 (0.077)	0.171 (0.078)
NLW	0.019 (0.004)	0.025	0.738	0.112	0.616	**0.051** ^4^ (0.010)	0.160 (0.211)
PFEC	5.348 (0.665)	5.344	−0.050	−0.001	0.152	0.138	**0.257** (0.029)

^1^Genetic (co)variances modified based on bending theory (Hayes and Hill, 1981).

^2^Estimated heritabilities are provided on the diagonal (in bold), estimated genetic correlations are provided above the diagonal, and bent genetic correlations are provided below the diagonal. Standard errors of estimated parameter values are shown in parentheses.

^3^TW, kg; WWT, kg; PFEC, (eggs/g)^1/3^.

^4^For bent parameter values, the heritability of NLB and NLW was 0.092 and 0.069, respectively.

The index coefficients and accuracies, and the genetic responses in goal traits, were then estimated using the bent genetic parameter values (Table 1) and the estimated phenotypic parameter values (Table 2).

**Table 2.** Estimates of phenotypic ($\sigma_{p}^{2}$) variances and correlations^1^

Trait^2^	$\sigma_{p}^{2}$	WWT	NLB	NLW	PFEC
TW	119.488 (1.279)	0.240 (0.035)	0.480 (0.007)	0.893 (0.002)	−0.050 (0.078)
WWT	11.790 (0.118)		0.115 (0.071)	0.091 (0.082)	−0.036 (0.073)
NLB	0.340 (0.003)			0.838 (0.007)	0.024 (0.015)
NLW	0.370 (0.003)				0.018 (0.018)
PFEC	20.810 (0.427)				

^1^Standard errors of the estimated parameter values are shown in parentheses.

^2^TW, kg; WWT, kg; PFEC, (eggs/g)^1/3^.

### Selection index coefficients

Index coefficients (***b***_***G***_) were found where the selection criteria were EBV (Schneeberger et al., 1992) as


$$\begin{array}{l} b_{G}=G_{11}^{-1}G_{12}a \end{array}$$

where $G_{11}$ is the *n* ×* n* genetic (co)variance matrix among the *n* selection criteria, $G_{12}$ is the *n* × 2 matrix of genetic (co)variances between the selection criteria and the 2 goal traits (TW and PFEC), and **a** is the 2 × 1 vector of scaled economic values for goal traits. The value of *n* is 3 for the benchmark scenario (WWT, NLW, and NLB) and 4 once PFEC is added as a selection criterion.

When selection criteria were phenotypes, index coefficients (***b_P_***) were found as:


$$\begin{array}{l} b_{P}=P_{11}^{\text{-}1}G_{12}a \end{array}$$

where $P_{11}$ is the *n* × *n* phenotypic (co)variance matrix among *n* selection criteria, with $G_{12}$ and **a** defined as before.

### Response in goal traits

When using EBV as selection criteria, annual genetic responses ($R_{a_{j_{EBV}}}$) in TW and PFEC were predicted as:


$$\begin{array}{l} R_{{\text{a}}_{j_{EBV}}}=i\frac{\left[\frac{b_{G}\prime G_{j}}{\sqrt{b_{G}\prime G_{12}a}}\right]}{L} \end{array}$$

where *i* is the average selection intensity of males and females, $G_{j}819G_{j}$ is a 1 x *n* vector of genetic (co)variances between goal trait *j* and the selection criteria, and *L* is the generation interval. The other vectors (***b_G_***, **a**) and matrix ($G_{12}$) are defined as earlier.

When phenotypic selection criteria were used, genetic responses ($R_{a_{j_{P}}}$) in the 2 goal traits were predicted as:


$$\begin{array}{l} R_{{\text{a}}_{j_{\text{P}}}}\;=i\frac{\left[\frac{b_{P}\prime G_{j}}{\sqrt{b_{P}\prime P_{11}b_{P}}}\right]}{L} \end{array}$$

with ${\text{\textbackslash{}textit b}}_{P}$ and ***P***_11_ defined as previously.

The rate of genetic response in TW was expressed in kilogram per year. Response of PFEC was expressed as the annual percent change (%/yr) from the average cube root transformed value of PFEC observed in Katahdin sheep. This transformation allowed an otherwise exponential distribution of PFEC to be approximated by a normal distribution. The average PFEC was 2,089 eggs/g as used by NSIP for hair sheep. In determining the average selection intensity, retention rates of 3% for rams and 26% for ewes were assumed, corresponding to selection intensities of 2.27 and 1.25, respectively. The generation interval was 3.5 yr, similar to that found by Nilson et al. (2024) in Katahdin sheep.

### Index accuracies

When EBV were used as selection criteria, index accuracies ($r_{HI_{EBV}}$) were calculated as:


$$\begin{array}{l} r_{{\text{HI}}_{\text{EBV}}}=\frac{b_{G}\prime G_{12}a}{\sqrt{\left(b_{G}\prime G_{11}b_{G}\right)\left(a\prime C_{22}a\right)}} \end{array}$$

where ***C***_22_ is the 2 × 2 genetic (co)variance matrix among goal traits (Table 1). The other variables are defined as earlier. When calculating an index using EBV as selection criteria, it is assumed that the values of ***G***_11_ are known without error. However, as noted by Schneeberger et al. (1992), in practice EBV would not be predicted with complete certainty, and differences in inbreeding among animals undoubtedly would exist. The index accuracies calculated, therefore, were viewed as their upper bounds when assuming breeding values were predicted perfectly.

When phenotypes were used as selection criteria, index accuracies ($r_{HI_{p}}$) were predicted as:


$$\begin{array}{l} r_{{\text{HI}}_{\text{p}}}=\frac{b_{P}\prime G_{12}a}{\sqrt{\left(b_{P}\prime P_{11}b_{P}\right)\left(a\prime C_{22}a\right)}} \end{array}$$

where variables are defined as earlier. This was viewed as the lower bound on accuracy since mass selection based only on phenotypic records of the animals themselves is a less accurate form of selection.

### Simulation and ranking of selection candidates

Breeding values for the selection criteria (WWT, NLB, NLW, and PFEC) were simulated using R 4.3.2 (R Core Team, 2023). A Cholesky decomposition was performed on the genetic (co)variance matrix (***G***_11_) to create breeding values for each trait. For each animal, a vector of 4 independent random values, expressed in SD units, was created by repeatedly sampling a normal distribution with mean 0 and variance 1. That vector was multiplied by the decomposed ***G***_11_ matrix to create the correlated breeding values for the 4 traits simulated for each animal. This process was repeated 200 times to create a simulated set of animals that varied in their breeding values for each trait. Our choice of the number of animals simulated was to reflect the size of a lamb crop from a moderately large NSIP Katahdin flock. Half of the animals were randomly assigned to be male, and the other half as female. Using their simulated breeding values, index scores ($I_{EBV}$) for the 11 indexes were obtained for the individual animals using:


$$\begin{array}{lll} I_{EBV}= & \;100+[b_{G_{WWT}}\left(BV_{WWT}\right)+b_{G_{NLB}}\left(BV_{NLB}\right)\; & +b_{G_{NLW}}\left(BV_{NLW}\right)+b_{G_{PFEC}}\left(BV_{PFEC}\right)] \end{array}$$

where $b_{G_{i}}$ represented the index coefficient associated with the breeding value ($BV_{i}$) for trait *i* for the given index.

Similar methods were applied when simulating phenotypic selection. The only difference was that a Cholesky decomposition of matrix ***P***_11_ was taken to simulate phenotypes for the selection criteria. Index scores ($I_{p}$) were obtained as:


$$\begin{array}{lll} I_{p}= & \;100+[b_{P_{WWT}}\left(P_{WWT}\right)+b_{P_{NLB}}\left(P_{NLB}\right)\; & +b_{P_{NLW}}\left(P_{NLW}\right)+b_{P_{PFEC}}\left(P_{PFEC}\right)] \end{array}$$

where *b*_Pi_ represented the index coefficient associated with the phenotype (*P*_*i*_) for an animal for the trait *i*. Like EBV, the phenotypes were expressed as deviations from their trait means of zero.

Animal rankings on the various index scores were compared using Spearman rank correlations and Jaccard similarities (Jaccard, 1901; R Core Team, 2023). Jaccard similarity coefficients (*J*) were calculated as:


$$\begin{array}{l} J=\;\;\;\frac{I_{benchmark,j}}{T_{benchmark,j}}\;\times 100 \end{array}$$

where $I_{benchmark,j}$ is the number of identically retained males or females between the benchmark and the *j*th index, and $T_{benchmark,j}$ is the total number of unique males or females that are retained between the benchmark and *j*th index.

As an alternative approach to Jaccard similarities, differences between the animals selected as replacements in the benchmark index and other indexes were calculated by counting the number of animals identically selected between the benchmark and a given index and then dividing by the total number of selected animals in the benchmark index.

When assessing the concordance among animals selected from the various indexes, the same retention rates—3% for rams and 26% for ewes—as used to predict genetic gains were used. The simulation was repeated 100 times and average Spearman rank correlation, Jaccard similarity coefficient, and proportion of identically selected animals were calculated. The SE of the repeated runs of the simulation was also obtained.

## Results

### Response in TW

**Figure 1.**
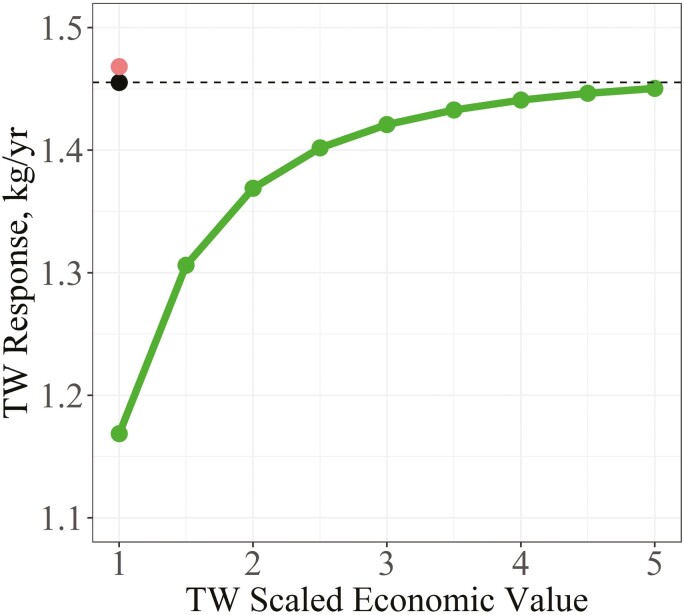
Response in TW between different indexes using EBV as selection criteria as the scaled economic value (**EV**) of TW was increased. The indexes compared were a benchmark index (black dot) where PFEC was excluded as both a selection criterion and as a goal trait (EV = 0), an index where PFEC was only included as a selection criterion (red dot), and an index where PFEC was included as both a selection criterion and goal trait (EV = −1) (green dots and line).

Under the benchmark scenario in which EBV were used as selection criteria, and PFEC was excluded as a selection criterion and with no contribution to the breeding objective (EV = 0), the predicted response of TW was 1.46 kg/yr (Fig. 1). After PFEC was added as a selection criterion, but still not included in the breeding objective, the predicted TW response was slightly improved to 1.47 kg/yr. Once PFEC was included as both a selection criterion and a goal trait with a scaled economic value of −1, the predicted TW response was reduced relative to the benchmark, though less so as the scaled economic value of TW increased from +1 to +5. Response in TW increased from 1.17 kg/yr when the scaled economic value of TW was +1 to 1.45 kg/yr when the scaled economic value of TW was +5. However, the increase was curvilinear with 98% of the maximum response in TW (1.42 kg/yr) achieved at a scaled economic value of + 3 for TW.

**Figure 2.**
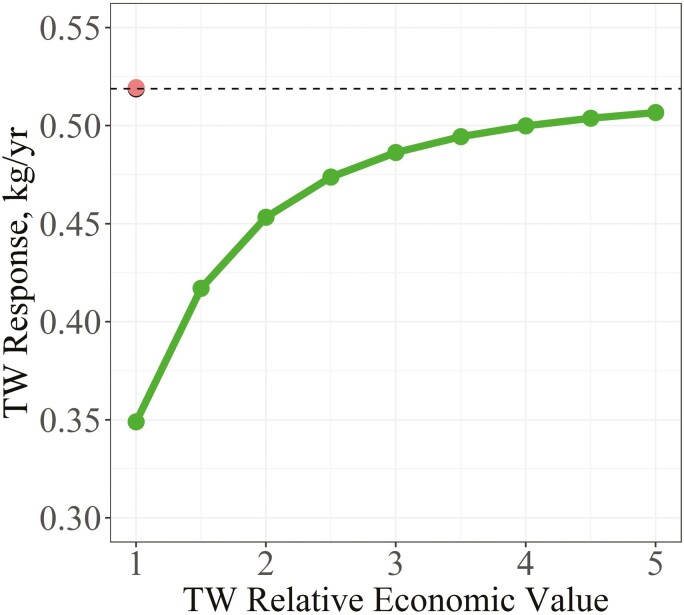
Response in TW between different indexes using phenotypes as selection criteria as the scaled economic value (**EV**) of TW was increased. The indexes compared were a benchmark index (black dot) where PFEC was excluded as both a selection criterion and as a goal trait (EV = 0), an index where PFEC was only included as a selection criterion (red dot), and an index where PFEC was included as both a selection criterion and goal trait (EV = −1) (green dots and line).

When using phenotypes as selection criteria, the predicted response for TW was 0.52 kg/yr under the benchmark scenario (Fig. 2). Adding PFEC as a selection criterion, but not as a goal trait, did not substantially change the predicted response in TW. Once PFEC was included as both a selection criterion and goal trait again at a scaled economic value of −1, response in TW increased from 0.35 kg/yr when the scaled economic value of TW was +1 to 0.51 kg/yr when the scaled economic value of TW was +5. Like with EBV as criteria, response in TW increased in a curvilinear fashion as its scaled economic value was increased and achieved 94% of its maximum response (0.49 kg/yr) at a scaled economic value of +3.

### Response in PFEC

**Figure 3.**
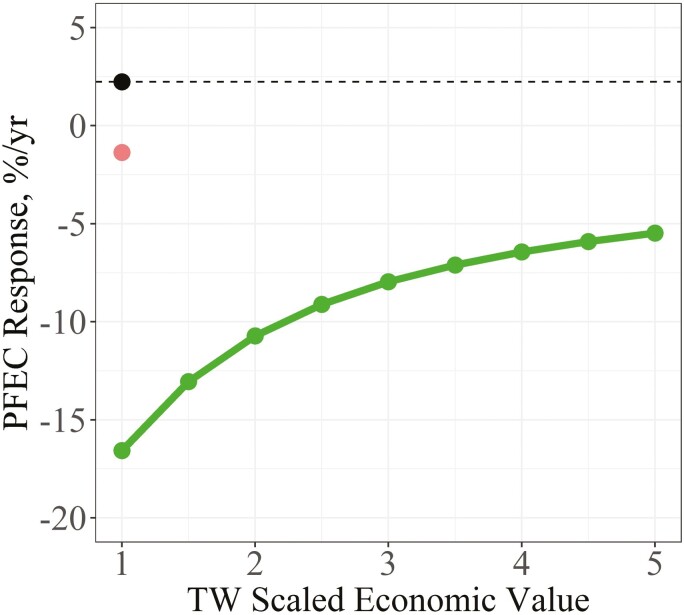
Response in PFEC between different indexes using EBV as selection criteria as the scaled economic value (**EV**) of TW was increased. The indexes compared were a benchmark index (black dot) where PFEC was excluded as both a selection criterion and as a goal trait (EV = 0), an index where PFEC was only included as a selection criterion (red dot), and an index where PFEC was included as both a selection criterion and goal trait (EV = −1) (green dots and line).

For the benchmark scenario with EBV-based criteria, and where PFEC was neither a selection criterion nor a goal trait (EV = 0), the predicted response for PFEC was +2.24%/yr (Fig. 3). When PFEC was added as a selection criterion, but not as a goal trait, its predicted response decreased to −1.38%/yr, which was favorable. Once PFEC was included as both a selection criterion and goal trait (EV = −1), its predicted response was greater when the scaled economic value for TW was +1 (-16.57%/yr) but declined curvilinearly to −7.96%/yr when the scaled economic value for TW was +3 and to −5.47%/yr when its values was +5.

**Figure 4.**
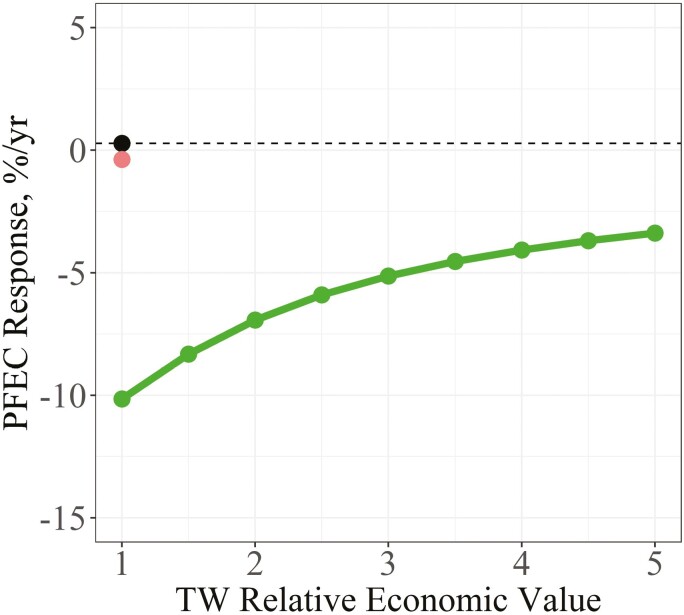
Response in PFEC between different indexes using phenotypes as selection criteria as the scaled economic value (**EV**) of TW was increased. The indexes compared were a benchmark index (black dot) where PFEC was excluded as both a selection criterion and as a goal trait (EV = 0), an index where PFEC was only included as a selection criterion (red dot), and an index where PFEC was included as both a selection criterion and goal trait (EV = −1) (green dots and line).

When using phenotypes as selection criteria, the predicted response for PFEC was +0.28%/yr under the benchmark scenario (Fig. 4). Adding PFEC to the selection criteria resulted in a PFEC response of −0.38%/yr. After also including PFEC as a breeding objective trait (EV = −1), response in PFEC ranged from −10.15%/yr when the scaled economic value of TW was +1 to −3.38%/yr when its value was +5. The same curvilinear pattern in genetic response was observed as the scaled economic value of TW increased. At a scaled economic value for TW of + 3, the response in PFEC was −5.13%/yr.

When the selection criteria were either EBV or phenotypes, an appropriate choice for the scaled economic value for TW appeared to be + 3 given a scaled economic value of −1 for PFEC. The genetic standard deviation of TW was 2.93 while that for PFEC was 2.31 for their bent parameter values (Table 1). For an index designed with a scaled economic value of + 3 for TW, the product of that value and genetic standard deviation for TW and PFEC were 8.79 and −2.31, respectively. This implies that approximately 79% of the emphasis in the breeding objective was on TW for this index.

### Index accuracies

With EBV as selection criteria, the index accuracy was 0.98 under the benchmark scenario (Table 3). Once PFEC was added to the breeding objective, index accuracies increased slightly to 0.99 regardless of the scaled economic value for TW. These large accuracies (approaching 1) were anticipated as TW was the sum of the weights of lambs weaned, directly reflected by 2 of its criteria (WWT and NLW). Also, when PFEC was added as a goal trait, since it was also a selection criterion, it was accurately predicted.

**Table 3.** Index accuracies when EBV or phenotypes are used as selection criteria

		Index accuracy	
TW scaled economic value	PFEC scaled economic value	EBV as criteria	Phenotypes as criteria
+1.0	0^1^	0.986	0.352
+1.0	0^2^	0.995	0.352
+1.0	−1	0.997	0.414
+1.5	−1	0.996	0.388
+2.0	−1	0.996	0.375
+2.5	−1	0.996	0.367
+3.0	−1	0.995	0.363
+3.5	−1	0.995	0.360
+4.0	−1	0.995	0.358
+4.5	−1	0.995	0.357
+5.0	−1	0.995	0.356

^1^PFEC not used as a selection criterion or goal trait (benchmark index).

^2^PFEC included as a selection criterion but not a goal trait.

When using phenotypic selection, the index accuracy was 0.35 under the benchmark scenario (Table 3). After including PFEC in the breeding objective, index accuracies decreased from 0.41 when the scaled economic value of TW was +1 to 0.36 when the scaled economic value of TW was +5. This decrease was due to PFEC, a trait in both the breeding objective and selection criterion, receiving less emphasis in the breeding objective as the scaled economic value of TW was increased.

### Ranking of selection candidates

Spearman rank correlations between index scores for the 200 simulated animals are provided in Table 4. The benchmark index in which PFEC was excluded from both the criteria and goal was used as the baseline for comparison. As the relative emphasis on PFEC increased, which coincided with reduced scaled economic values for TW, the correlations between indexes and the benchmark became weaker. The correlations among the index scores based on phenotypic criteria were smaller than those based on EBV criteria when the scaled economic value of TW was less than + 4. That tendency likely was due to the larger (co)variances among phenotypes as compared to EBV criteria.

**Table 4.** Spearman rank correlations between benchmark index^1^ and other indexes using either EBV or phenotypes as selection criteria, and their SE

		Spearman correlation with benchmark index	
TW^2^ scaled economic value	PFEC^3^ scaled economic value	EBV (SE)	Phenotypic (SE)
+1.0	0^2^	1.000	1.000
+1.0	0^3^	0.989 (0.000)	0.998 (0.000)
+1.0	−1	0.689 (0.004)	0.613 (0.005)
+1.5	−1	0.806 (0.003)	0.753 (0.003)
+2.0	−1	0.864 (0.002)	0.832 (0.002)
+2.5	−1	0.897 (0.002)	0.878 (0.002)
+3.0	−1	0.918 (0.001)	0.907 (0.001)
+3.5	−1	0.931 (0.001)	0.927 (0.001)
+4.0	−1	0.941 (0.001)	0.940 (0.001)
+4.5	−1	0.948 (0.001)	0.950 (0.001)
+5.0	−1	0.953 (0.001)	0.958 (0.001)

^1^Benchmark index is where PFEC is excluded from both the selection criteria and goal.

^2^PFEC not used as a selection criterion or goal trait (benchmark index).

^3^PFEC included as a selection criterion but not a goal trait.

As less emphasis was placed on TW (reduced scaled economic values), both the Jaccard similarity coefficients and proportions of identically selected ewes and rams between the benchmark and other indexes consistently decreased (Tables 5–8). With the higher selection intensity in rams, that discordance was higher than that in ewes.

**Table 5.** Estimated proportion of selected lambs in common with the benchmark index^1^ when using EBV as selection criteria, and their SE

TW^2^ scaled economic value	PFEC^3^ scaled economic value	Proportion of males in common (SE)	Proportion of females in common (SE)
+1.0	0^2^	100	100
+1.0	0^3^	85 (1.9)	92 (0.4)
+1.0	−1	33 (2.5)	61 (0.7)
+1.5	−1	46 (2.6)	69 (0.7)
+2.0	−1	54 (2.4)	75 (0.6)
+2.5	−1	59 (2.2)	78 (0.6)
+3.0	−1	64 (2.1)	80 (0.6)
+3.5	−1	66 (1.9)	82 (0.6)
+4.0	−1	67 (2.0)	83 (0.6)
+4.5	−1	67 (2.0)	84 (0.5)
+5.0	−1	69 (2.0)	85 (0.5)

^1^Benchmark index is where PFEC is excluded from both the selection criteria and goal.

^2^PFEC not used as a selection criterion or goal trait (benchmark index).

^3^PFEC included as a selection criterion but not a goal trait.

**Table 6.** Estimated Jaccard similarity coefficients between the benchmark index^1^ and all other indexes when using EBV as selection criteria, and their SE

TW scaled economic value	PFEC scaled economic value	Male Jaccard similarities (SE)	Female Jaccard similarities (SE)
+1.0	0^2^	100	100
+1.0	0^3^	78 (2.6)	86 (0.7)
+1.0	−1	23 (2.1)	44 (0.7)
+1.5	−1	34 (2.6)	53 (0.8)
+2.0	−1	41 (2.5)	60 (0.8)
+2.5	−1	46 (2.4)	64 (0.8)
+3.0	−1	50 (2.4)	67 (0.8)
+3.5	−1	52 (2.3)	69 (0.8)
+4.0	−1	53 (2.3)	72 (0.8)
+4.5	−1	54 (2.4)	73 (0.8)
+5.0	−1	57 (2.5)	74 (0.8)

^1^Benchmark index is where PFEC is excluded from both the selection criteria and goal.

^2^PFEC not used as a selection criterion or goal trait (benchmark index).

^3^PFEC included as a selection criterion but not a goal trait.

**Table 7.** Estimated proportion of selected lambs in common with the benchmark index^1^ when using phenotypes as selection criteria, and their SE

TW scaled economic value	PFEC^3^ scaled economic value	Proportion of males in Common (SE)	Proportion of females in common (SE)
+1.0	0^2^	100	100
+1.0	0^3^	94 (1.3)	98 (0.3)
+1.0	−1	28 (2.5)	56 (0.8)
+1.5	−1	39 (2.7)	65 (0.7)
+2.0	−1	46 (2.9)	72 (0.6)
+2.5	−1	54 (3.0)	77 (0.6)
+3.0	−1	61 (2.6)	80 (0.6)
+3.5	−1	63 (2.5)	82 (0.6)
+4.0	−1	66 (2.5)	84 (0.5)
+4.5	−1	69 (2.5)	86 (0.5)
+5.0	−1	72 (2.4)	87 (0.5)

^1^Benchmark index is where PFEC is excluded from both the selection criteria and goal.

^2^PFEC not used as a selection criterion or goal trait (benchmark index).

^3^PFEC included as a selection criterion but not a goal trait.

**Table 8.** Estimated Jaccard similarity coefficients between the benchmark index^1^ and all other indexes when using phenotypes as selection criteria, and their SE

TW scaled economic value	PFEC scaled economic value	Male Jaccard similarities (SE)	Female Jaccard similarities (SE)
+1.0	0^2^	100	100
+1.0	0^3^	92 (1.9)	95 (0.5)
+1.0	−1	19 (1.9)	39 (0.8)
+1.5	−1	29 (2.4)	49 (0.8)
+2.0	−1	35 (2.7)	56 (0.8)
+2.5	−1	43 (3.0)	63 (0.8)
+3.0	−1	49 (2.9)	67 (0.8)
+3.5	−1	51 (2.8)	70 (0.8)
+4.0	−1	54 (2.9)	73 (0.8)
+4.5	−1	58 (3.0)	75 (0.8)
+5.0	−1	62 (2.9)	77 (0.8)

^1^Benchmark index is where PFEC is excluded from both the selection criteria and goal.

^2^PFEC not used as a selection criterion or goal trait (benchmark index).

^3^PFEC included as a selection criterion but not a goal trait.

## Discussion

Gastrointestinal parasites annually cost the global sheep industry tens of billions of dollars in anthelmintic pharmaceuticals alone (Roeber et al., 2013; Charlier et al., 2020). In the past, GIN infection has been controlled primarily through anthelmintics. However, because of increases in anthelmintic resistance among GIN, and concerns among consumers regarding the use of pharmaceuticals in livestock production, alternative genetic solutions are desirable (Jackson and Coop, 2000; Howell et al., 2008; Busch et al., 2020; Newman et al., 2020).

Parasitic infection has harmful effects on economically important traits such as wool quality and quantity, growth rate, carcass weight, and survival rate (Beck et al., 1985; Kelly et al., 2014). In southern India, animals grew 0.008 kg/d more slowly for every unit increase in eggs/g (Ilangopathy et al., 2019). In western Australia, lambs from a population selected for lower FEC were 1.6 kg heavier at weaning than lambs from an unselected population. In the same study, unselected lambs required anthelmintic treatment 15% to 17% more often (Greeff and Karlsson, 2020). In another study (Fthenakis et al., 2005), ewes with GIN challenge that were not treated near parturition birthed smaller lambs, produced less milk, and, consequently, weaned lighter lambs. Though GIN pose an ever-rising problem to the global sheep industry, their management must still occur in tandem with the many other priorities of sheep producers.

In this study, we predicted genetic gains from increasing the relative economic value of TW in our breeding objective whilst holding the weight on PFEC constant in a series of indexes. Our supposition was that by comparing those gains, we could identify an index with more favorable outcomes. An alternative approach could have been a desired gains index (Kempthorne and Nordskog, 1959; Cunningham et al., 1970; Gibson and Kennedy, 1990), in which the change in one trait, such as PFEC, is set as a multiple of the change in another trait, such as TW. However, we did not have a clear view a priori on a desired balance between the goal traits. We, therefore, chose a more exploratory approach in which different scenarios and their consequences were tested.

PFEC was used as a selection criterion instead of FEC recorded at weaning at around 60 d of age. This was because of its higher heritability and larger genetic (additive) variance (Ngere et al., 2018). However, FEC at weaning is also an attractive selection criterion and goal trait. It has been shown to have a stronger negative correlation with weaning and postweaning weights than PFEC (Ngere et al., 2018).

In defining an index for hair sheep producers that includes GIN resistance, scaled economic values must be set in a way that balances reducing PFEC with maintaining adequate gains in TW. Presently, producers selecting animals solely on the U.S. Maternal Hair Index are potentially selecting animals with higher PFEC. With studies attributing substantial economic loss to GIN (Nieuwhof and Bishop, 2005; Qamar et al., 2011; Charlier et al., 2020), and wide interest from producers to reduce GIN through management and genetic selection, it appears that the current makeup of the U.S. Maternal Hair Index does not capture the full scope of priorities amongst hair sheep breeders. Currently, NSIP breeders who wish to select for GIN resistance must do so separately from TW, which may lead to less optimal genetic responses in both traits. If they wish to appropriately balance GIN resistance with TW, PFEC must be added to the breeding objective. However, TW directly affects income and has a longstanding tradition as a selection priority. Therefore, TW should still receive a majority of the selection emphasis.

The selection indexes constructed in this study used either EBV or phenotypes as selection criteria. Since breeding values were assumed to be known without error, index accuracies and responses in goal trait represented an upper bound on predicted outcomes when using EBV as selection criteria. When phenotypes were used, those accuracies and responses characterized a lower bound on predicted outcomes since a single phenotypic record on an individual itself was viewed as one of the simplest forms of selection. Realized accuracies and genetic responses would lie somewhere in between these bounds. Therefore, with the use of an index with scaled economic values of +3 and −1 for TW and PFEC, respectively, we would anticipate annual genetic gains of between 0.49 and 1.42 kg/yr for TW and between −5.13% and −7.96% for PFEC.

When PFEC was added to the benchmark index as a selection criterion, its response went from positive to negative, which was favorable. This change was due to the slightly negative genetic correlation between TW and PFEC; selection to increase TW thereby resulted in a correlated response (reduction) in PFEC. Also, the predicted TW response increased slightly numerically. This indicated that at the very least, PFEC should be added to the index as a selection criterion since improvement is achieved for both traits relative to the benchmark. However, many hair sheep producers likely would prefer to see even greater reductions in PFEC than those observed in an index where it was only included as a selection criterion.

Once PFEC was included in the breeding goal (scaled economic value of −1), reducing the scaled economic value of TW much below +3 did not appear to provide the desired results. The TW responses decreased substantially and at an increasing rate as its scaled economic value decreased. When its value was +1, 80% and 67% of its benchmark response was retained when using EBV and phenotypes as selection criteria, respectively. Though reductions in PFEC were substantial with this index, the predicted loss in TW gain would likely be unacceptable to most producers. However, if future environmental conditions cause an increase in GIN infection levels, or if anthelmintic resistance becomes increasingly endemic, a scaled economic value of TW of less than + 3 may become more attractive. Links between GIN infection and increased spring, autumn, and winter temperatures have been observed in the U.K. (van Dijk et al., 2008). Since the rise in temperatures is a global phenomenon (Moss et al., 2008), GIN resistance will likely receive more emphasis in future indexes.

Constructing a new index with a scaled economic value for TW and PFEC of +3 and −1, respectively, may currently be the most sensible approach. This index appeared to provide a reasonable balance of reducing PFEC with only small losses in gain in TW. At these scaled economic values, with EBV as the selection criteria, 98% of gains in TW responses were retained relative to the benchmark index. When phenotypes were the selection criteria, 94% of the gains were retained. Genetic response in PFEC switched from increasing to favorably decreasing by 8% and 5%, respectively, when EBV and phenotypes were the selection criteria in the index.

Additionally, by not reducing the scaled economic value of TW below +3, re-ranking among selection candidates was limited. Such is positive because 1) producers are often more accepting of smaller changes in their breeding decisions, 2) negative consequences for producers who do not record PFEC are likely minimal, and 3) any potential criticism from seedstock producers whose animals have changed index rankings is lessened. Collectively, this would likely increase the rate of adoption of the new index. Using EBV as selection criteria, 64% of the male and 80% of the female selection candidates were identical to those in the benchmark index. Using phenotypes as the selection criteria, 61% of the male and 80% of the female selection candidates remained identical to those in the benchmark index. Though there were some differences, seedstock and/or commercial producers may be comfortable with this extent of change.

Placing limited emphasis on PFEC was also consistent with the design of the New Zealand Maternal Index. In that index, only 13% of the selection emphasis was placed on FEC while lamb survival and weight received 51% and 30% of the selection emphasis, respectively (Santos et al., 2015; Zhang and Amer, 2021). Though the production systems in New Zealand differ from those in the U.S., it reaffirms the idea that parasitic infection affects costs and returns but is by no means the primary driver of profits.

Accuracies did not change substantially between the indexes tested. Thus, it was not a major consideration when determining the proper balance among scaled economic values. However, when using EBV as selection criteria, the index accuracies approaching 1 suggest that the selection criteria were reliable predictors of TW and PFEC and explain most of their genetic variation. Such is not surprising, as PFEC was used as a selection criterion and TW is the product of the average WWT of lambs in a litter and the NLW.

If implemented, adding PFEC to the breeding objective for hair sheep in the U.S. could change the genetic trajectory of selection from less to more GIN resistance while still achieving strong genetic gains in TW. Although the scaled economic value combination of + 3 and −1 for TW and PFEC, respectively, seems reasonable, the impact of such an index on net profit is hard to define. Albeit the loss of small, slower genetic gains in TW would seemingly reduce income. Reduced morbidity due to GIN resistance and lower production costs including less use of anthelmintic pharmaceuticals, may, however, compensate for such losses. Additionally, in environments where the GIN burden is high, the predicted 2% loss in TW response may not be observed as the gains in parasite resistance may counter losses in weight gain in lambs. Genotype by environment interactions, which have been reported for body weights and FEC in Katahdin lambs (Arisman et al., 2023b), may contribute to such outcomes.

Even though setting the scaled economic value of TW at + 3 and PFEC at −1 may be acceptable to many producers, there will be breeders who are unenthusiastic about adding PFEC to the breeding objective. Some existing hair sheep enterprises have little to no GIN infection due to climate or management, and therefore see little benefit to record or select for PFEC. With this being the case, subindexes could be provided to allow producers greater flexibility in their selection emphasis (Amer et al., 2001). Amer et al. (1998) created 2 subindexes, terminal and maternal, that were derived from a total index that included both terminal and maternal traits. The terminal subindex was created by setting the economic values of some maternal traits in the total index to zero. A similar approach may be useful for U.S. hair sheep breeders, where the scaled economic value for PFEC can be adjusted to meet a producer’s unique production goals. Producers with extensive grazing systems and located in hot and humid environments could place more relative emphasis on PFEC in their breeding objective, while producers with intensive production systems or flocks with low GIN infection levels could place no emphasis on PFEC (scaled economic value of zero). However, regardless of the importance of PFEC in the breeding objective, incorporating PFEC as a selection criterion could encourage producers to record PFEC phenotypes to predict TW more accurately.

Although defining true economic values for TW and PFEC would be desirable, this remains a challenge due to the many environmental and managerial factors that interact with PFEC. Parasite abundance often varies between flocks and years, which makes it hard to delineate GIN resistance from low or variable exposure levels. Even within a year, parasite abundance within a flock can vary as temperature, rainfall, and forage abundance fluctuate (Fritsche et al., 1993; Mir et al., 2008). Also, GIN load in one year may be influenced by management in the previous year (Amer et al., 1999). Another complication is the variety of deworming strategies used, with salvage, tactical, and strategic deworming the most common (Cruz-Rincon, 2020). Since these strategies differ in timing and the extent of GIN challenge necessary to justify treatment, their costs of application differ. Additionally, the efficacy of anthelmintics is uncertain as parasites develop drug resistance (Bath, 2006). With many of these complexities yet to be characterized in the U.S. sheep industry, the approach of defining scaled economic value used in this study provides a solution to an otherwise difficult problem.

There is interest in improving other heritable traits not presently found in the U.S. Maternal Hair Index, such as ewe udder conformation and longevity. However, adding PFEC as a goal trait offers a next step for including a fitness trait in the breeding objective since industry-wide phenotypic recording and genetic evaluation of this trait are already underway. Additionally, as has been illustrated, PFEC can also be included in the breeding objective in a manner that allows for continuing genetic gain in TW while also improving flock GIN resistance.

In the NSIP genetic evaluation, EBV for DWWT and MWWT rather than WWT are available as selection criteria. Therefore, prior to implementing this index, the possible consequences of using those alternative criteria on the choice of the scaled economic values is needed. Such an analysis would determine if the scaled economic value of + 3 and −1 for TW and PFEC, respectively, remains most favorable.

## Conclusions

In the absence of formally derived economic values for PFEC, sensible scaled economic values for TW and PFEC were + 3 and −1, respectively. With these scaled economic values, approximately 79% of the emphasis in the breeding objective was on TW. With this balance, TW retained 98% of its benchmark response while PFEC were reduced by 8% annually. This is in contrast to the predicted increase in PFEC when it was excluded as both a selection criterion and goal trait. Such an index, with low to moderate emphasis on PFEC, should be well received among producers because it addresses their desire for improving GIN resistance while maintaining strong genetic gains in TW, the trait that directly impacts income. Furthermore, including producers in deciding the scaled economic values for TW and PFEC used in practice could build excitement and increase the implementation of the index. Work remains. Defining economic values via a bioeconomic model would be desirable to ensure that the index maximizes profitability within a given production system. Furthermore, prior to implementing this index, research into the consequences of parsing WWT into its components (DWWT and MWWT), as done in the NSIP genetic evaluation, on the design of the index is needed. However, by incorporating PFEC into a revised index, genetic progress in both traits can clearly be achieved, with a positive impact on both the productivity and fitness of hair sheep.

## Abbreviations

DWWT: direct weaning weight
EBV: estimated breeding values
FEC: fecal egg count
GIN: gastrointestinal nematode
MWWT: maternal weaning weight
NLB: number of lambs born
NLW: number of lambs weaned
NSIP: National Sheep Improvement Program
PFEC: postweaning fecal egg count
TW: total weight of lamb weaned per ewe lambing
WWT: weaning weight
